# Bibliometric analysis of global endometriosis research, 2002 to 2021: A review

**DOI:** 10.1097/MD.0000000000035723

**Published:** 2023-11-24

**Authors:** Siyao Deng, Dongxia Yang, Qiyao Li, Haoran Dai, Weikang Tang, Lihuang Lu, Jing Liu, Xiuhong Wu

**Affiliations:** a Country Academy of Pharmacy, Heilongjiang University of Chinese Medicine, Harbin, Heilongjiang, China; b Department of Gynecology Medicine, Second Affiliated Hospital of Heilongjiang University of Chinese Medicine, Harbin, Heilongjiang, China.

**Keywords:** bibliometrics, CiteSpace, endometriosis, VOSviewer

## Abstract

Endometriosis is a common disease of reproductive-age women and an important cause of dysmenorrhea and infertility. Information on endometriosis is complex and there is a lack of summarization of available results. The study aims to evaluate the overall distribution of publications related to endometriosis to provide a foundation for further research. The Web of Science Core Collection was searched for articles published in the field of endometriosis. Our survey revealed the structure, hotspots, and development trends of endometriosis-related research and publications.

## 1. Introduction

Endometriosis is a common disease of women of childbearing age, with a prevalence of 5% to 10%. Endometriosis is an important cause of dysmenorrhea and infertility, which negatively affect the quality of life and physical and mental health of women.^[1]^ Treatment of endometriosis is difficult; treatment usually involves surgery and drugs, and relapse occurs frequently. The annual worldwide cost of treatment is estimated to exceed 20 billion US dollars, a great burden for society and patient families.^[2]^

Research and journal publications on endometriosis have increased dramatically, and the large number of articles has brought many new directions and ideas for development of medical treatment. However, few attempts have been made to systematically analyze the evolution of scientific results of endometriosis research. At the same time, the academic literature regarding endometriosis continues to expand, which makes it increasingly difficult to focus on particular areas and institutions, and possible interconnections and gaps between disciplines.

Bibliometric analysis maps the knowledge domain of academic literature and can address the aforesaid challenge by identifying current trends, research networks, and related topics.^[3,4]^ In this report, we review VOSviewer and CiteSpace bibliometric methods for use in endometriosis research. Our goal was to obtain fruitful visual information on the global scientific output of endometriosis research, to help readers better understand research progress, and to encourage new researchers to seize the frontier of the field. This report will help new researchers comprehend current research on endometriosis and the investigators and institutions active in the research. We also report which journals are more likely to see reports on endometriosis and keyword outbreak of articles, which can help readers identify research trends.

## 2. Materials and methods

The literature data used in this study were downloaded from the Web of Science Core Collection on October 21, 2022, which is composed of the following databases: Science Citation Index Expanded, 1990-present, Social Sciences Citation Index, 1983-present, Arts & Humanities Citation Index, 1983-present, Conference Proceedings Citation Index-Science, 1996-present, Conference Proceedings Citation Index-Social Science & Humanities, 1996-present, Emerging Sources Citation Index, 2017-present, Current Chemical Reactions, 1985-present, and Index Chemicus, 1993-present. Online retrieval was performed using keyword *endometriosis*. The time span was set to 2002 January 1st through 2021 December 31st. The publication type was not limited. For each publication, authors, keywords, institutions, and cited references were downloaded.

VOSviewer is a software tool for constructing and visualizing bibliometric networks. The VOSviewer constructs a graphical representation of bibliometric data to understand a research field in an easily interpretable way.^[5]^ Some results may provide reference for possible interdisciplinary studies. In the density visualization picture, the more items in the vicinity of a point, the greater the weight of adjacent items, and the color of the point will be closer to yellow. Conversely, the smaller the number of items in the vicinity of a point, the lower the weight of adjacent items, and the closer the color of the point will be to blue. CiteSpace is another information visualization software tool used to visualize trends and mutations of keywords in a specified period of time. For this paper, VOSviewer 1.6.18 was used to analyze the number of articles published by authors, institutions, and countries, and journal co-citation and literature co-citation. CiteSpace 5.7.R2 was used to make a timeline diagram of synthetic keywords. GraphPad Prism 9 was used to construct histograms to visualize the number of publications on endometriosis during the aforesaid 20-year period.

## 3. Results

### 3.1. Annual publication trend

**Figure 1.**
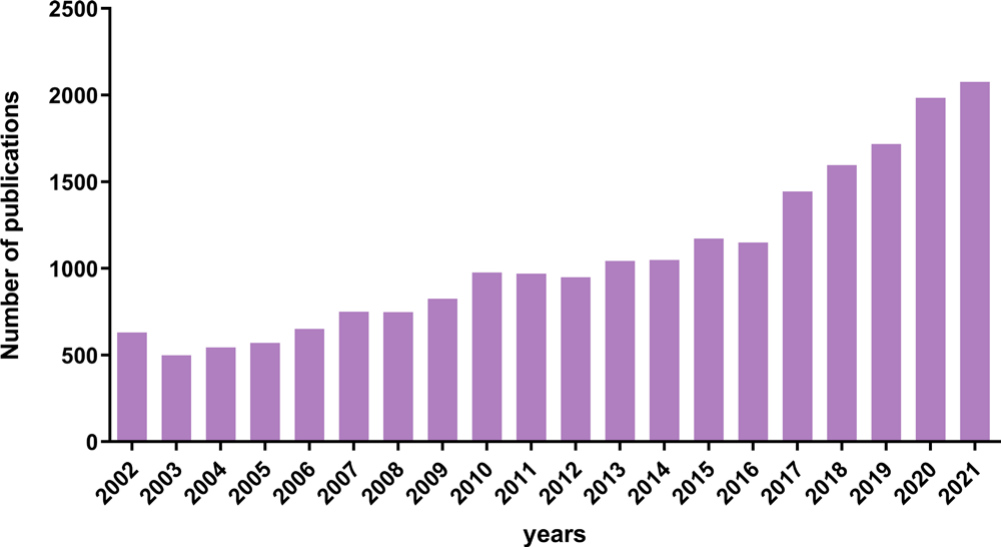
Annual number of endometriosis publications.

**Figure 2.**
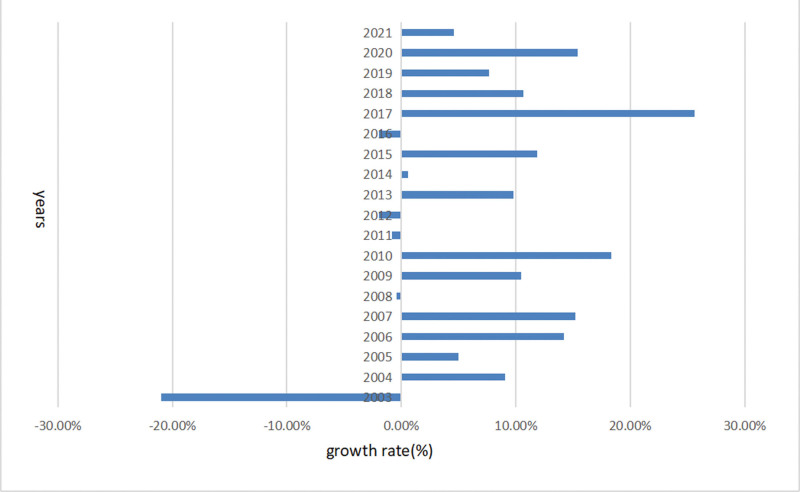
Annual growth rate of publications on endometriosis.

As shown in Figure 1, we identified 21,336 articles about endometriosis published in the 20 years from 2002 through 2021. Beginning in 2013, the number of articles published yearly exceeded 1000. We used Microsoft Excel^®^ to draw the growth rate chart (Fig. 2). Before 2017, the number of publications increased slowly; most of the annual number of endometriosis publications represented a positive growth trend; a small part of the number of articles was negative growth but the negative value was small. In general, the number of publications rose slowly, but the growth rate in 2017 was 25.59%, the highest of all years. After 2017, all years were in a growth state. In 2021, the number of published papers reached 2075.

### 3.2. Publication analysis

**Figure 3.**
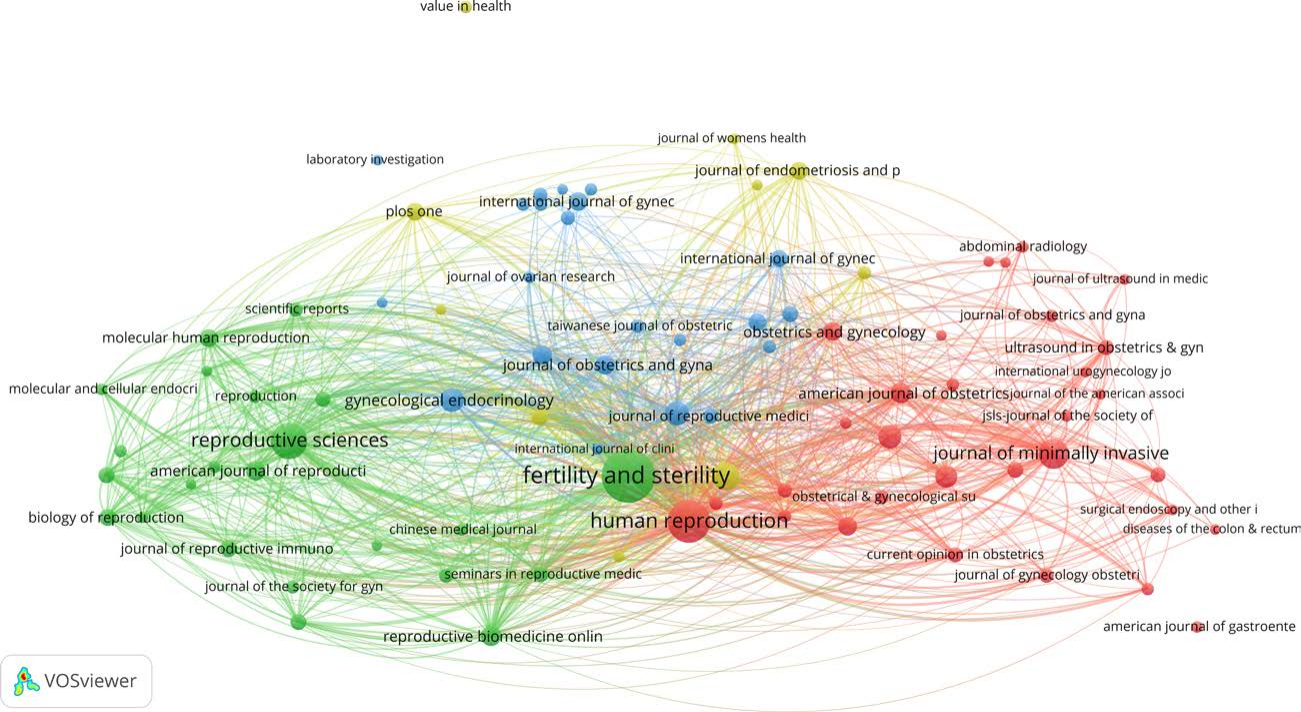
The journal citation network of endometriosis related publications.

The 21,336 articles from 2002–2021 were published in 2313 journals. Table 1 lists the top 10 journals based on number of publications. Five of the top 10 journals had an IF > 3. The top 2 journals, *Fertility and Sterility* and *Human Reproduction*, had IF of 7.49 (2021) and 6.353 (2021), respectively. Those 2 journals contributed 3518 articles in 20 years, 16.49% of all the articles. Four of the top 10 most frequently cited journals are in Table 1, namely, *Fertility and Sterility, Human Reproduction, European Journal of Obstetrics Gynecology and Reproductive Biology*, and *Journal of Minimally Invasive Gynecology*. The journal citation synthesis network (Fig. 3).

**Table 1:** The top 10 journals for number of endometriosis publications.

Rank	Journal	2021 IF	5-yr IF	Total
1	Fertility and Sterility	7.49	8.109	2215
2	Human Reproduction	6.353	7.736	1303
3	Reproductive sciences	2.924	3.103	860
4	Journal of Minimally Invasive Gynecology	4.314	3.935	544
5	European Journal of Obstetrics & Gynecology and Reproductive Biology	2.831	2.778	460
6	Gynecological Endocrinology	2.277	2.237	321
7	Archives of Gynecology and Obstetrics	2.493	2.804	306
8	Geburtshilfe und Frauenheilkunde	2.754	2.438	273
9	Bjog an International Journal of Obstetrics and Gynaecology	7.331	7.37	248
10	Reproductive Biomedicine Online	4.567	4.603	201

IF = Impact Factor, the frequency with which articles in a particular journal are cited in a particular year or period. IF is an important measure of the influence of an academic journal. The 5-yr IF is not simply the average of the impact factors of the prior 5 yr. The 5-yr data are used in the calculation of the impact factor instead of the 2-yr data of the general impact factor. Because the 5-yr impact factor encompasses the data of 5 yr, to a certain extent, the impact factor of some journals fluctuates greatly due to the particularly high citation numbers of 1 or 2 articles. Nevertheless, the 5-yr impact factor may better reflect the recent average level of the journal compared with 2-yr impact factor (2 yr).

### 3.3. Country, institution, and author analysis

**Figure 4.**
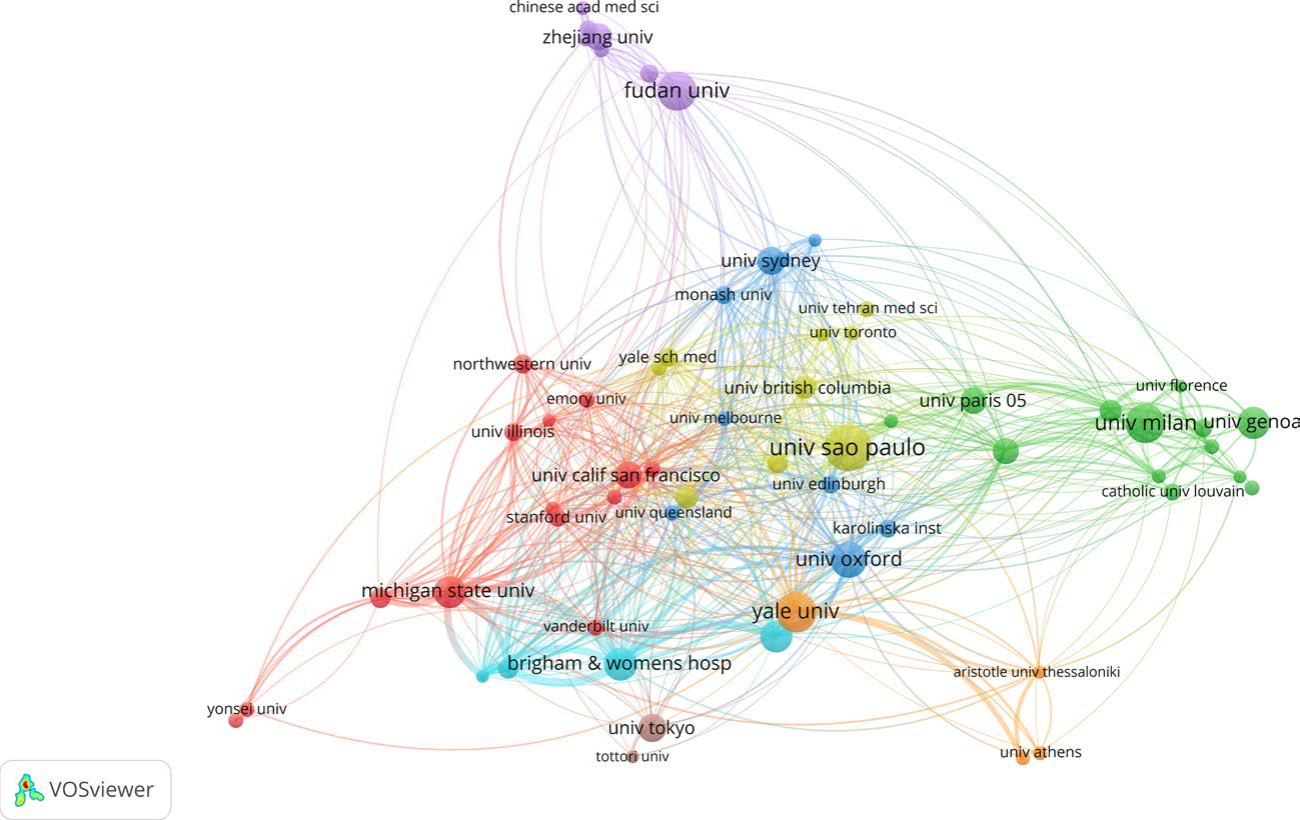
Institution network of endometriosis-related publications.

The 21,336 endometriosis articles were from 126 countries and regions and included 10,096 institutions. The United States had the largest article contribution (24.23%), well ahead of other countries. China was the second highest contributing country, with less than half the number of articles compared with the United States. Five of the top 10 institutions with the largest number of publications (Table 2 were from the United States (1992 articles), 3 from France (1571 articles), 1 from Belgium (370 articles), and 1 from Brazil (356 articles). The United States accounted for 50% of the top 10 institutions. Figure 4 is the organization cooperation network graph. The connection between nodes represents the cooperation relationship between institutions, and the distance and thickness of the connection represent the degree of cooperation between institutions. The top institutions are also more closely related to other institutions.

**Table 2:** Top 10 productive countries for the study of endometriosis.

Rank	Country	Total	Proportion(%)
1	USA	5169	24.2
2	China	2118	9.9
3	Italy	2061	9.7
4	Japan	1377	6.5
5	England	1335	6.3
6	Germany	1267	5.9
7	France	1240	5.8
8	Brazil	883	4.1
9	Australia	852	4.0
10	Canada	736	3.4

**Figure 5.**
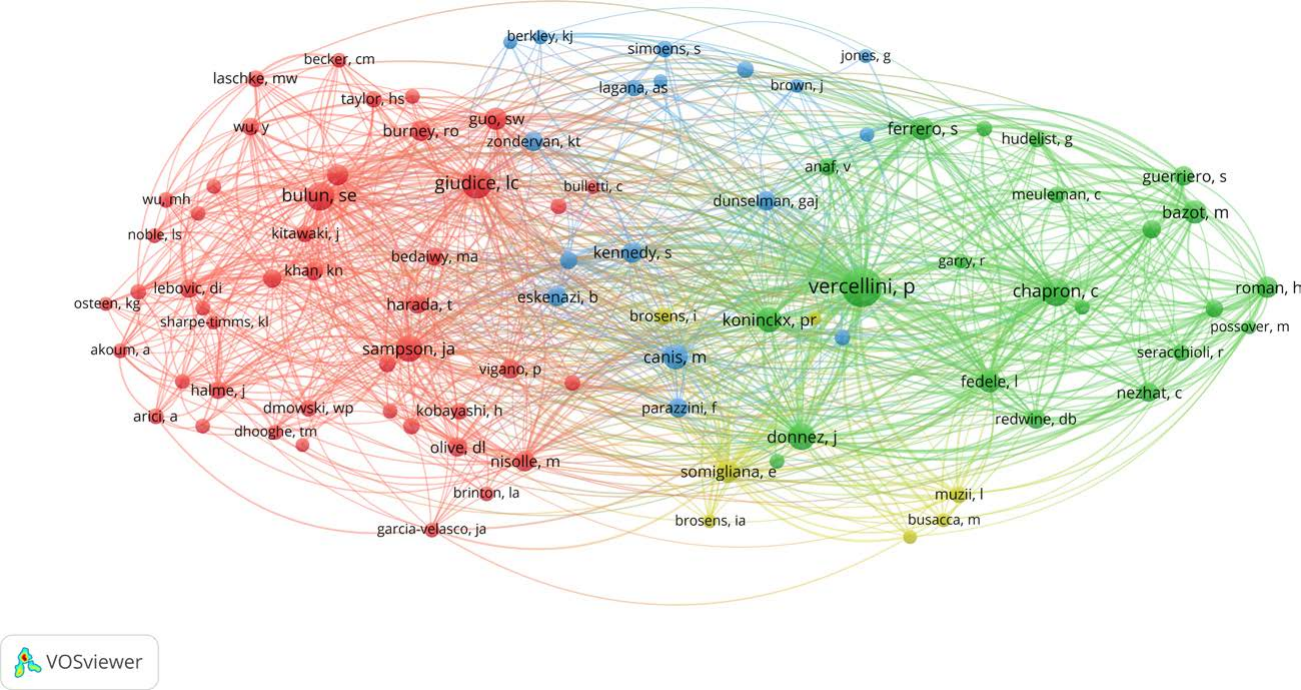
Co-citation author network of endometriosis-related publications.

There were 50,910 authors who contributed to the study of endometriosis. Four top 10 Authors were from Italy, 2 from France, 2 from the United States, 1 from Brazil, and 1 from China. These 10 authors contributed 1717 papers during the 20 years, which accounted for 8% of all endometriosis-related publications. It is worth noting that Vercellini P, ranked No. 4, and Somigliana E, ranked No. 5, collaborated to publish 158 studies on endometriosis. Both of these researchers are from Italy. Four of the authors in the ranking are from Italy, including Ferrero S whose publications are mostly in Q1 and Q2. Italy ranked third in the number of articles published, only 57 fewer than China, which ranked second. The remaining Authors also had more or less collaborative relationships, such as Chapron C(No. 2) and Mauricio SA (No. 8), who published 10 articles together in 20 years. Of these top 10 Authors, 5 were from 1 or more of the 10 institutions shown in Table 3. The results showed that the most frequently cited Authors accounted for 3 of the top 10 in the number of publications, namely Vercellini P, Chapron C and Ferrero S.The Author citation network diagram (Fig. 5).

**Table 3:** Top 10 productive institutions for the study of endometriosis.

Rank	Institution (country)	Total	Proportion(%)
1	Udice French Research Universities (France)	664	3.1
2	Assistance Publique Hopitaux Paris (France)	535	2.5
3	Harvard University (USA)	486	2.3
4	Yale University (USA)	419	2.0
5	University of California System (USA)	394	1.8
6	Universite Paris Cite (France)	372	1.7
7	Ku Leuven (Belgium)	370	1.7
8	Universidade De Sao Paulo(Brazil)	356	1.7
9	Harvard Medical School (USA)	328	1.5
10	Brigham Womens Hospital (USA)	295	1.4

The color of the node represents the different categories, the size of the node represents the number of nodes, and the line between the nodes represents the connection between the nodes (Figs. 3–5).

### 3.4. Reference co-citation and journal co-citation analysis

**Figure 6.**
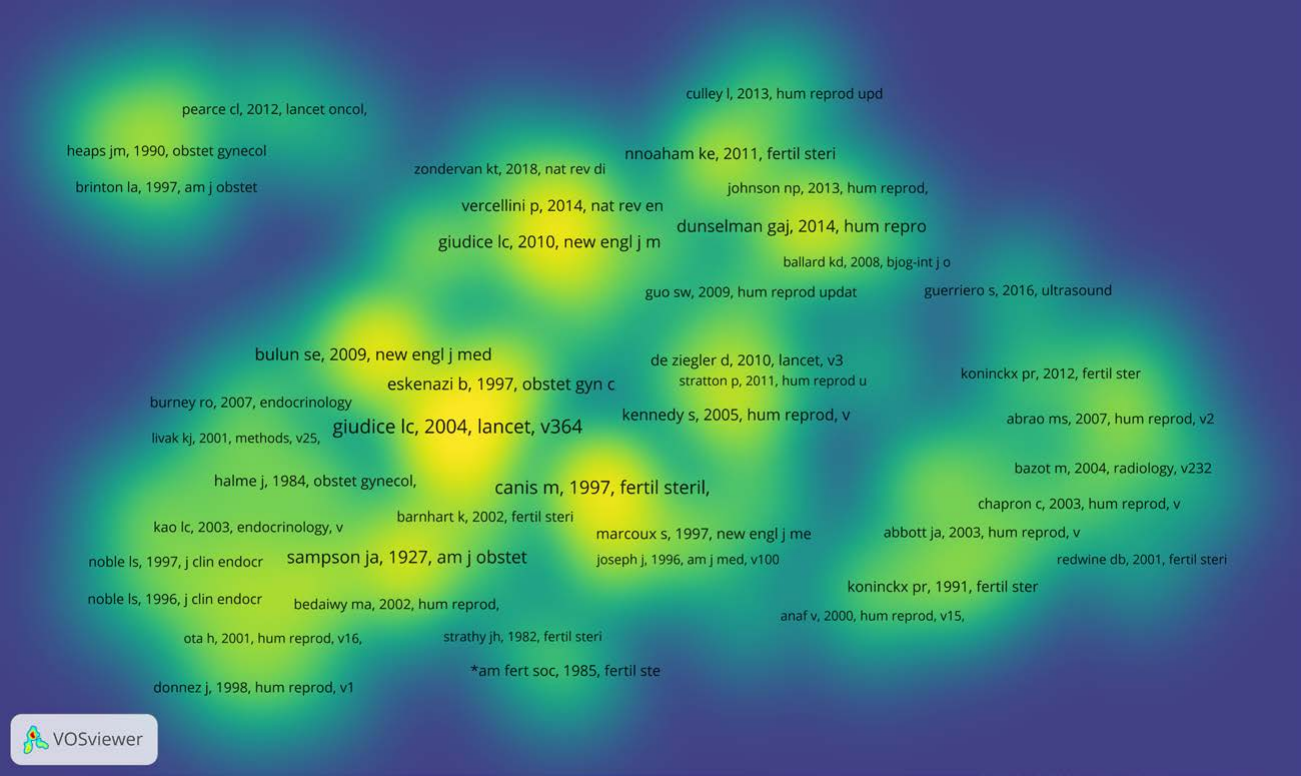
Co-citation cited reference density of endometriosis related publications.

**Figure 7.**
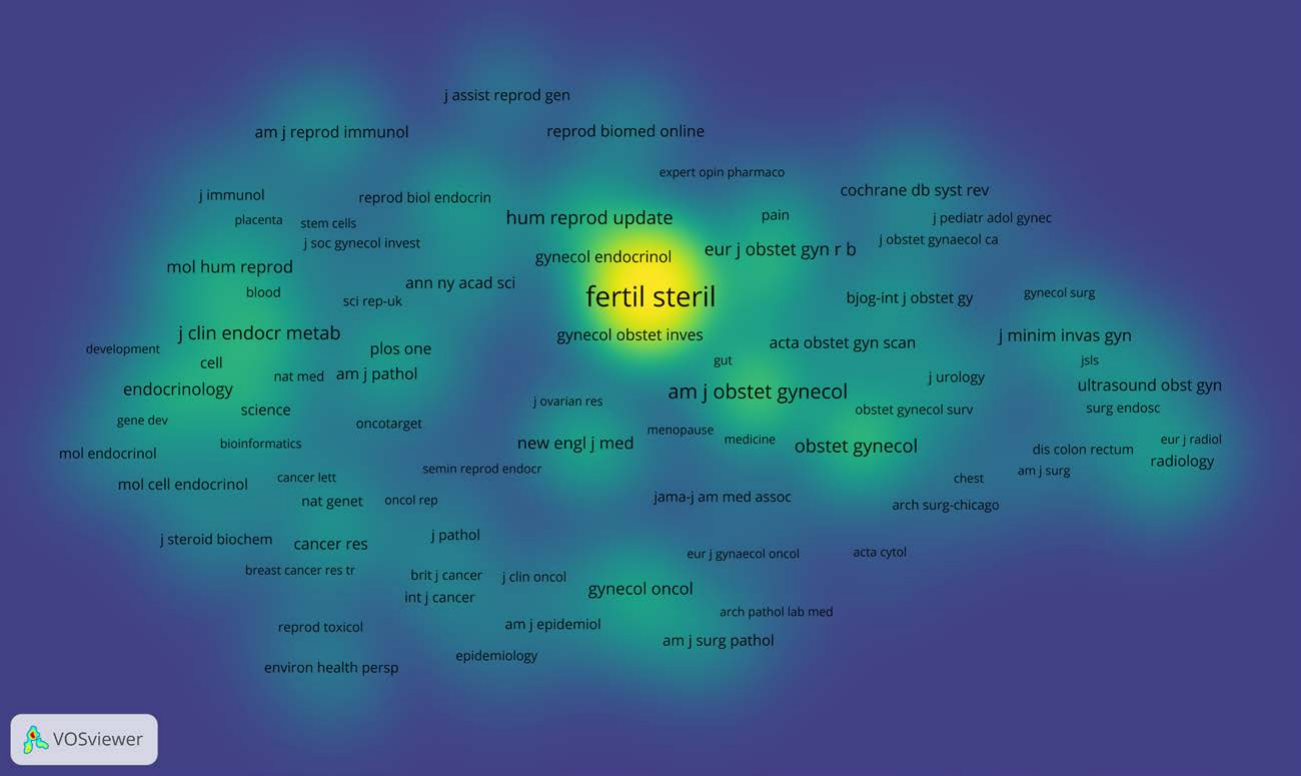
Co-citation cited journal density of endometriosis related publications.

Reference co-citation analysis and journal co-citation analysis can reveal clusters of relevant literature and journals that can be linked to scientific fields, and clusters that are close to each other in the map represent close relations. The most frequently cited article was “Endometriosis” published in Lancet in 2004, which was cited for 1897 times. The most cited journal was *Fertility and Sterility*, which was cited 70,883 times. Among the top 10 co-cited references and co-cited journals, 4 overlapped: *Fertility and Sterility, American Journal of Obstetrics and Gynecology, New England Journal of Medicine* and *Human Reproduction*. The 4 journals included 7 top 10 co-cited references. In addition, 2 of these 4 journals coincided with the most frequently published journal: *Fertility and Sterility* (ranked first) and *Human Reproduction* (ranked second). About references co-cited and journals co-cited, most of the references were cited more articles is a system of endometriosis is introduced, including the pathogenesis, clinical diagnosis, and disease-related factor of contact. These highly cited references show that a thorough understanding of the theoretical knowledge is essential before in-depth study of the disease. The reference co-citation (Fig. 6) and journal co-citation (Fig. 7).

There are 3 colors in the picture, blue, green and yellow. The larger the number of items, the closer to yellow, followed by green, followed by blue (Figs. 6 and 7).

### 3.5. Keyword analysis

**Figure 8.**
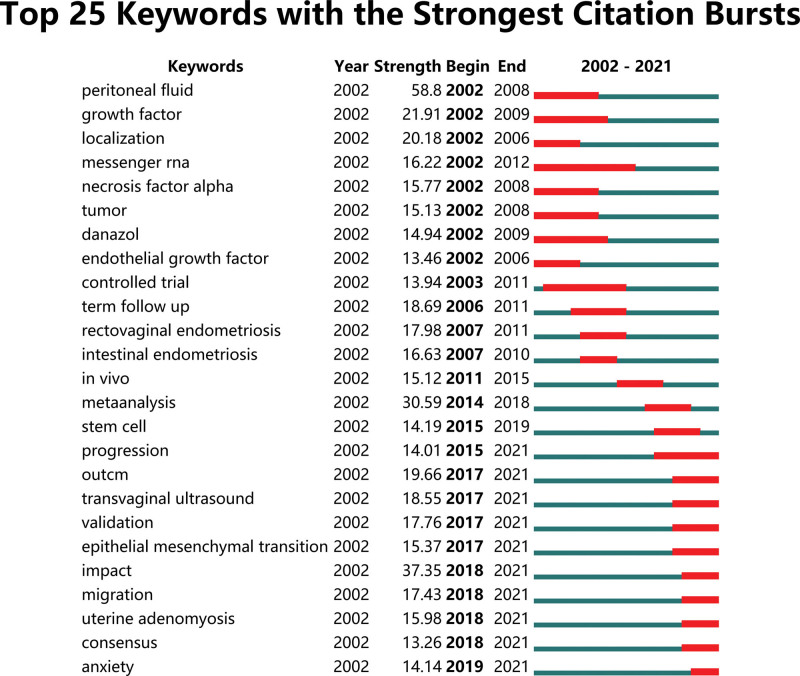
Top 25 keywords with the strongest citation bursts.

We used CiteSpace 5.7.R2 to capture the research frontier keywords with the strongest citation outbreak from 2002 through 2021. Keywords are the core summary of a paper, and analysis of keywords can provide an overview of the topic of a paper. The top 25 keywords were screened according to the outbreak intensity (Fig. 8). The green line represents the 20-year period, and the red line indicates the timeline from the beginning to the end of the outbreak of the keyword. The words *peritoneal fluid* burst most (58.8). Subsequent research is subjected to meta-analyses of various diagnoses. Meta-analysis is used to collect, sort, and analyze many studies performed for a particular topic and to seek therefrom a clear relation between the problem or the variables concerned. A meta-analysis can make up for the deficiency of a traditional review article. There is no fixed format nor protocol for the writing of traditional reviews, and there is no uniform standard for evaluating the quality of included studies. The quality of traditional reviews is greatly affected by the experience of the authors, the breadth of data collection, and the quality of the included literature; in addition, the total effect size of interventions cannot be quantitatively analyzed. Different authors who study the same particular issue are likely to reach completely different conclusions. The words with the greatest burst in the prior decade were *impact, outcome, transvaginal ultrasound, validation*, and *migration*.

## 4. Discussion

Worldwide, 10% of women of reproductive age, more than 176 million women total, experience endometriosis^[6]^; the incidence of dysmenorrhea is 40% to 60%, low fertility is 21% to 47%, and/or pelvic pain is 71% to 87%.^[7]^ Endometriosis not only affects the quality of life, but also it poses a heavy economic burden on society.

Bibliometrics, in a scientific and feasible manner, can display visually the massive amount of literature and present the highly active research topics and trends by cluster analysis and the construction and drawing of a network atlas.^[8]^ Bibliometric analysis methods can provide a comprehensive understanding of a field, which is of great help to subsequent research and, in the case of human studies, clinical application. For this paper, we used VOSviewer and CiteSpace to analyze the articles on endometriosis published in the prior 20 years and to examine the current development of endometriosis research.

We found 21,336 related articles in the Web of Science Core Collection database for bibliometric analysis and visualization. From 2002 to 2012, the number of articles published yearly was hundreds, and from 2013 to 2021, the yearly number increased to more than 1000. Since 2017, the number of publications on endometriosis has been in a positive growth state each year, which indicates that research on endometriosis is in a rising period. Especially in recent years, attention has been focused on the topic, and treatment and pathogenesis are being studied intensively.

In terms of publications, *Fertility and Sterility* and *Human Reproduction* were the 2 most common journals, with 2215 and 1303 articles, respectively. The impact factors of these 2 journals are 7.49 and 6.353. Endometriosis is an inflammatory, estrogen-dependent disease associated with pelvic pain and infertility.^[9]^ We often find that “endometriosis” is related to “infertility”; thus, it is easy to see the literature related to endometriosis in journals that address fertility and infertility.

This inconsistency led us to speculate that because the number of institutions that publish is large enough, and the proportion of all institutions in the world is large enough, more research will naturally be produced. We found that the authors with large numbers of reports also published more review papers than experimental research papers; thus, new researchers can understand the theoretical knowledge of endometriosis from these reviews.

In terms of keywords, at that time, studies on peritoneal fluid focused mainly on the determination of hormone levels and inflammatory factors therein. For example, peritoneal fluid confirmed that the expression of the estrogen receptor in macrophages was related to macrophage activation, which also supported the theory that the estrogen receptor was involved in development and persistence of endometriosis by acting on macrophages in the peritoneal cavity.^[10]^ The importance of Interleukin-8(IL-8) in the pathogenesis of endometriosis was reinforced by the detection of IL-8 in peritoneal fluid.^[11]^ Suggested that Interleukin-17(IL-17) has an important function in the pathogenesis of early endometriosis and endometriosis-related infertility.^[12]^ The increased concentrations of hepatocyte growth factor, Interleukin-6(IL-6), and estradiol in peritoneal fluid demonstrate the combined activities of these cytokines and ovarian steroids in the production of hepatocyte growth factor from endometrial tissues in active endometriosis.^[13]^

The term *mRNA* was important for ten years (2002–2012). Endometriosis literature related to mRNA is generally concerned with determination of enzymes, such as matrix metalloproteinase^[14]^ and P450.^[15]^ Messenger RNA can also be used for the detection of signal pathways, such as glucocorticoid, eicosanoid,^[16]^ and sphingosine pathways.^[17]^

*Epithelial mesenchymal transition, transvaginal ultrasound, meta-analysis, migration*, and *anxiety* have been frequent keywords. In the first decade of endometriosis research, investigators tended to focus on the expression and measurement of various cytokines, whereas the second decade seems to have a focus on disease diagnosis and impact. Often, endometriosis is not diagnosed in time, mistaken for other diseases, or overlooked for the best opportunity for treatment. With considerable advances in diagnostic imaging such as transvaginal ultrasound and magnetic resonance imaging, exploratory laparoscopy should no longer be used to diagnose endometriotic lesions.^[18]^ Transvaginal ultrasound has become the main diagnostic method for pelvic endometriosis and adenomyosis.^[19]^

Some questions addressed by a meta-analysis of endometriosis research are the following: Accuracy of ultrasonography in the diagnosis of endometriosis in different locations.^[20]^ Critical analysis of the value of transvaginal ultrasound in noninvasive, pre-operative diagnosis of enteral endometriosis.^[21]^ Review of the diagnostic accuracy of transvaginal ultrasound in the preoperative detection of uterosacral ligaments in patients with clinical suspicion of deep infiltrating endometriosis.^[22]^ Advances in diagnostics have transformed invasive diagnoses into noninvasive tests, greatly improving the patient experience. Epithelial-mesenchymal transition is integral in development, wound healing and stem cell behavior, and pathologically promotes fibrosis and cancer progression.^[23]^ *Migration* is also closely related to the development of cancer. Although endometriosis is a benign disease in terms of histomorphology, it has the same biological behaviors of invasion, implantation, and adhesion as the behaviors of malignant tumors; in fact, some endometriosis patients will incur malignant transformation.^[24]^ Therefore, it is possible to further study the pathways or targets involved in metastasis.

Our analysis had limitations. The selection of a single database made it impossible for us to include more data. There was no unified standard for the setting of software algorithms and parameters.

## 5. Conclusion

Our research makes up for the absence of a comprehensive summary of endometriosis literature. We analyzed 20 years of global endometriosis research and its trends. Our analysis can shape the direction of further research and provide new points of view for solutions to a prominent women health problem.

## Author contributions

**Conceptualization:** Xiuhong Wu, Qiyao Li, Lihuang Lu.

**Data curation:** Haoran Dai.

**Methodology:** Weikang Tang.

**Supervision:** Xiuhong Wu.

**Validation:** Lihuang Lu.

**Visualization:** Jing Liu.

**Writing – original draft:** Siyao Deng.

**Writing – review & editing:** Dongxia Yang.
